# Emerging Breakthroughs in Nano-Ginseng Innovations and Their Therapeutic Implications in Type 2 Diabetes

**DOI:** 10.3390/ph19010186

**Published:** 2026-01-21

**Authors:** Pragya Tiwari, Kyeung-Il Park, Sayanti Mandal

**Affiliations:** 1 Department of Horticulture and Life Science, Yeungnam University, Gyeongsan 38541, Republic of Korea; 2 Department of Chemistry and Biochemistry, Sharda School of Basic Sciences & Research, Sharda University, Greater Noida 201306, India; mandalsayanti@gmail.com

**Keywords:** drug-delivery systems, ginsenosides, herbal therapeutics, Korean red ginseng, nanoformulations, nanotized herbal drugs, pharmacokinetics

## Abstract

**Background/Objectives:** Diabetes is characterized by multiple metabolic disorders, defined by high blood sugar levels over a prolonged duration. Type 2 diabetes (T2D) comprises defective insulin secretion, its ineffective utilization, or both, resulting in hyperglycemia. The disease is one of the leading causes of mortality, according to the WHO, and necessitates the development of advanced therapeutics. **Methods:** This systematic review was conducted in accordance with the PRISMA guidelines. The study and execution of the literature review followed a timeframe of 3–6 months, during which the conceptualization, execution, analysis, writing, and editing were conducted. Ginsenosides, triterpenoids from the *Panax* genus, are widely recognized for their promising antidiabetic effects, mediated through mechanisms that include glucose uptake, insulin secretion, antioxidant activity, and anti-inflammatory pathways. Ongoing clinical trials in patients with IGT or Type 2 diabetes have shown an improvement in insulin sensitivity and glucose control, and consolidate the therapeutic potential of ginseng pharmacotherapy. **Results:** This viewpoint summarizes the most recent discoveries on the hypoglycemic mechanisms of ginsenosides for Type 2 diabetes and its associated complications, with a major focus on ginseng-based drug development. An emphasis is placed on how ginsenosides control blood glucose levels and regulate signaling pathways, investigating their antidiabetic mechanisms and potential. **Conclusions:** Preclinical studies suggest that nano-innovations in ginseng have the potential to address therapeutic challenges, improve systemic circulation, lower the toxicity of biomolecules, and improve bioavailability, defining exciting outcomes. Furthermore, well-designed human clinical trials are necessary to understand the antidiabetic mechanisms and pharmacological potential of ginseng and/or ginsenosides in drug development.

## 1. Introduction

### 1.1. Current Overview of Type 2 Diabetes Prevalence

Worldwide, one of the most common endocrine disorders is Type 2 diabetes, characterized by elevated blood sugar levels. The disease has raised severe concerns with increased prevalence and mortality rates worldwide. According to a recent report in *The Lancet*, the global prevalence of diabetes reached 529 million in 2021, with a higher prevalence documented in the Middle East and North Africa [1]. The recent findings indicate that more than 1.31 billion people are estimated to have diabetes by 2050, creating a huge health crisis and an economic burden, particularly for low- and middle-income countries [2]. Type 2 diabetes is characterized by insulin resistance, insulin deficiency, or a combination of both [3,4], which is further aggravated by lifestyle factors including obesity, a sedentary life, a high calorie intake, and increased insulin requirements due to elevated free fatty acids and insulin resistance [5]. Moreover, studies have suggested that increased free fatty acids and hyperglycemia cause oxidative stress and inflammation in Type 2 diabetes [6]. In Type 2 diabetes, insulin resistance is linked to lipid abnormalities, decreased HDL cholesterol levels, and increased VLDL and triglyceride levels [7]. Moreover, chronic hyperglycemia leads to multi-organ failure of the kidney, retina, heart, and nervous system [8,9], leading to the patient’s death.

In a quest for effective medication, drug development has witnessed unprecedented advances, with metformin developed as the first line of treatment. Other key antidiabetic therapeutics include GLP agonists (e.g., exenatide), sulfonylureas (e.g., glibenclamide), α-glucosidase inhibitors (e.g., acarbose), and dipeptidyl peptidase-4 inhibitors (e.g., sitagliptin), among others [10,11,12]. The popular drug class of biguanides (e.g., metformin) promotes peripheral glucose uptake by cells. Insulinotropic drugs such as sulphonylureas (e.g., glibenclamide) induce insulin secretion from pancreatic cells [13]. While these medications have demonstrated potent efficacy in glycemic regulation, the associated side effects include hypoglycemia, lactic acidosis, and gastrointestinal problems, which comprise major limitations. Furthermore, regular insulin injections can be painful and inconvenient for patients, while the use of long-term oral drugs may cause drug toxicities [14]. Recent therapies (GLP-1 RA and SGLT2) significantly improve the condition in Type 2 diabetes, in addition to providing stroke prevention (GLP-1 RA), fewer heart failure incidences, and a lowered risk of cardiovascular disease [15,16]. However, none of the currently available medications for Type 2 diabetes have been successful in providing a complete cure, highlighting the need for further investigation. In the current context, diabetes management primarily focuses on antidiabetic oral medications, dietary monitoring, and insulin analogs [17,18]. Gene therapy for diabetes management holds tremendous potential for a long-term cure through therapeutic delivery into specific cells, offering advantages over conventional pharmacotherapies [19]. Gene therapy targets insulin resistance, increases energy utilization, and improves glucose tolerance via multiple mechanisms [20,21]. The current therapeutic regimes focus on investigating and repurposing traditional herbal medicines and bioactive phytoconstituents to identify and screen new/novel drug-like candidates for disease treatment.

### 1.2. Herbal Medicines, Plant Secondary Metabolites, and Diabetes Management: Molecular Mechanisms and Nanotechnological Advances

The WHO has recognized the medicinal use of 21,000 plants worldwide, and 400 plant species have been documented for their antidiabetic effects [22,23]. Moreover, the World Ethnobotanical Inspection has reported the use of approximately 800 plants for the treatment of diabetes [24,25], attributed to their potent efficacy, comparatively safer profiles, and biocompatible nature compared to conventional medications [26]. While herbal medicines minimize the adverse effects caused by the conventional drugs, they are efficient scavengers of ROS and function as antioxidants [27]. Although many plant species demonstrate antidiabetic effects, only a small fraction have undergone clinical evaluation to validate their potential for drug development. The antidiabetic activity of the plant extract is due to the presence of specialized metabolites, including flavonoids, coumarins, phenolics, and terpenoids, displaying different molecular mechanisms: the inhibition of α-amylase, α-glucosidase, PTP1B, and PPARγ functions [28,29,30,31]; improvement in β-cells’ function; improved glucose uptake; and enhanced insulin secretion, similar to the oral antidiabetic drugs [22,32]. In addition, plant-based phytoconstituents are beneficial in alleviating the secondary complications associated with diabetes [33]. While plant-based medicines show potent efficacy in diabetes management, current challenges, including the poor pharmacokinetics and bioavailability of plant metabolites, necessitate further research.

A globally recognized plant for its multiple pharmacological attributes, *Panax ginseng* has been increasingly investigated for its potential in diabetes management. *Panax quinquefolius* (American ginseng) and *P. ginseng* (Asian ginseng) are the most widely studied plants in the genus, having been thoroughly investigated for their antidiabetic mechanisms and the efficacy of their specialized metabolites [34]. Furthermore, current research focuses on understanding the in vivo and in vitro mechanisms of ginseng in animal models and cell lines for Type 2 diabetes. Furthermore, ongoing preclinical studies on ginseng administration in human trials have shown promising outcomes [35]. Recent advances in ginseng biotechnologies suggest that ginsenosides have key prospects as antidiabetic drug candidates and necessitate further investigation. With substantial biotechnological progress in medicinal plant research, we can hope for feasible and sustainable solutions to the current therapeutic challenges associated with plant-based drugs.

Nanotechnological advances in medicinal plant research have witnessed unprecedented developments, particularly in the areas of drug discovery and management. Novel avenues in Type 2 diabetes management focus on nano-based herbal medicines, including the development of herbal nanoformulations and their targeted and precise delivery, to address the bottlenecks associated with conventional antidiabetic drugs [36,37]. In addition, the poor efficacy and pharmacokinetics of plant-based drugs can be improved by nano-carriers and herbal nanoformulations. Nano-sized herbal drugs (NHDs) address the poor intestinal permeability, reduced solubility, and poor retention upon oral administration [38,39,40]. The emerging breakthroughs highlight the increased recognition of nano-innovations for targeted plant-based nanoparticles (NPs) delivery, via nanorobots, nano-pumps, nanoemulsions, polymeric NPs, NLCs, and NHDs [41,42,43,44]. In diabetes and associated abnormalities, nanoformulations facilitate effective insulin delivery by oral administration, compared to parenteral preparations, and better patient compliance [45,46,47] (Table 1).

### 1.3. Objectives and Highlights of the Review

This state-of-the-art review discusses the expanding pharmacological scope and potential of ginsenosides in Type 2 diabetes and summarizes the recent advances in nano-innovations in ginseng biotechnologies for drug development. Nano-ginseng interventions have far-reaching implications, demonstrating improved therapeutic efficacy and pharmacokinetics, effective systemic circulation, improved bioavailability, and fewer side effects—a step towards achieving sustainable healthcare goals.

- To investigate the current nano-innovations in ginseng and the antidiabetic mechanisms of bioactive ginsenosides.
- To evaluate the emerging strategies and research gaps in the field.
- To provide feasible solutions to address the limitations associated with ginseng-based therapeutics for drug development.

While the current ongoing research promises translational success, further investigations are required to address existing bottlenecks in the pharmacokinetics of natural products and develop novel, effective nanoformulations for diabetes management.

## 2. Materials and Methods

### 2.1. Review Design and Methodology

This systematic review was conducted in accordance with the PRISMA guidelines [79]. The study and execution of the literature review followed a timeframe of 3–6 months, during which the conceptualization, execution, analysis, writing, and editing of the content were completed. Selection of the relevant information was made through a search across the electronic databases PubMed (https://pubmed.ncbi.nlm.nih.gov) (accessed on 15 August 2025) [80], Scopus (https://www.scopus.com/home.uri?origin=sbrowse) (accessed on 15 August 2025 [81], and Google Scholar (https://scholar.google.com) (accessed on 15 August 2025) [82], to retrieve the most relevant studies on Type 2 diabetes, *Panax* genus, *P. ginseng*, and nanobiotechnological interventions and their current challenges and prospects. A literature search was performed according to the following items: “*Panax*”, “ginseng”, “ginsenosides”, “antidiabetic”, and “nanobiotechnology” or “nanotechnology”. The literature was screened and compiled based on adherence to the following criteria: (a) peer-reviewed articles (original research, reviews, books, databases, conference abstracts, editorials, preprints) focusing on natural products for Type 2 diabetes, plant specialized metabolites and their antidiabetic mechanisms, and the emerging innovations in therapeutic development; and (b) studies on *P. ginseng*, precisely, the antidiabetic mechanisms of ginsenosides and their existing challenges and feasible and sustainable solutions. Information obtained from local and foreign books, as well as other sources (including websites and organizations), was also included. In total, (*n* = 330) records were identified, and duplicate records were removed (*n* = 48). The relevant literature was screened (*n* = 282), and irrelevant records were excluded (*n* = 55) (articles in which the outcomes were not of interest). Reports were sought after retrieval (*n* = 227), while some reports were not retrieved (*n* = 53). The exclusion criteria comprised: n1, articles with no full text available; n2, articles in other languages (non-English); and n3, articles excluded after abstract screening. After a comprehensive and exhaustive search, 180 studies/items of literature were shortlisted and compiled to frame the literature review.

### 2.2. Inclusion and Exclusion Criteria

**Figure 1:**
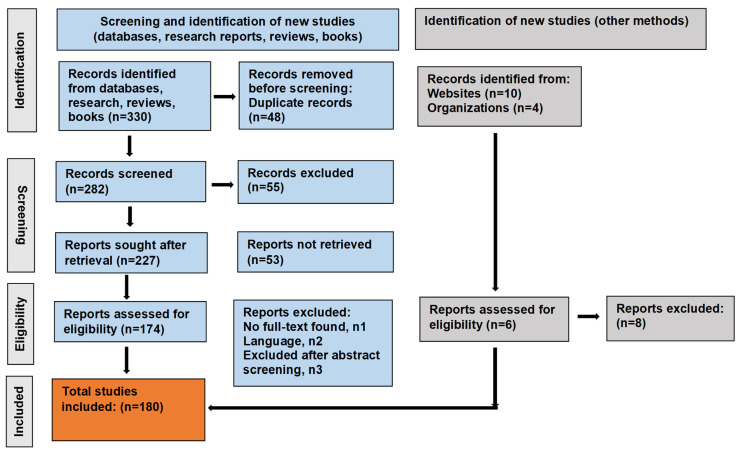
Study flow diagram.

The literature search comprised a period of publication (2003–2025) and was limited to articles in the English language. The preliminary search was focused on non-clinical studies, reports on the plants of the *Panax* genus, the triterpenoid ginsenosides, and their antidiabetic activity, traditional use, and recent progress in drug development for Type 2 diabetes. Preclinical studies in experimental animal models (mice and rats) and human cell lines to elucidate the antidiabetic mechanisms of ginsenosides were included. Studies comparing the effect of different ginsenosides with a control group (animals/cell lines treated with another substance) were considered. Published reports in non-indexed journals (before 2003) were not included. Moreover, studies on ginsenosides and plant extracts for other bioactivities, articles in other languages (except English), articles with no full text available, and those that were not found suitable after abstract screening (research duplication) comprised the exclusion criteria (Figure 1).

## 3. Results

***Panax* Genus and Therapeutic Implications in Type 2 Diabetes: Traditional Uses, Present Initiatives, and Healthcare Prospects**

Traditional records show a rich history of use in Korean, Chinese, and Japanese herbal medicine and in Western countries, driving increased research investigation of the genus *Panax* and its pharmacologically important metabolites, ginsenosides [83,84,85]. The pharmacological benefits of ginseng are attributed to the presence of ginsenosides, polyphenols, polysaccharides, volatile oils, and flavonoids, among other specialized metabolites [86,87]. To date, more than 200 ginsenosides have been reported from the *Panax* genus and heat-processed products derived from ginseng [34], and they are classified as oleanane and dammarane saponins. Universally, ginsenosides are classified in three categories: PPT-type, e.g., ginsenosides Re, Rg1, Rg2, Rf, and Rh1; PPD-type, e.g., ginsenosides Rb1, Rb2, Rb3, Rc, Rg3, Rh2, Rd, and compound K; and oleanolic acid-type (e.g., ginsenosides Ro, Ri, Rh3). The tetracyclic triterpene saponins possess a similar parent moiety and are represented by the PPT and PPD types, while pentacyclic triterpenoid saponins include the oleanolic-type ginsenosides. Studies have documented the importance of the tetracyclic triterpene moiety in conferring antidiabetic properties to PPT- or PPD-type ginsenosides [88]. In traditional medicine, the benefits of ginseng polysaccharides and saponins in managing diabetes and its associated complications are well-documented [89,90]. Ginseng roots have been utilized in anti-aging studies, chronic disease prevention, and health-improvement initiatives since ancient times [83]. The multiple antidiabetic mechanisms of ginsenosides include the regulation of glucose-lipid homeostasis, anti-inflammatory actions, improvement of insulin resistance, regulation of oxidative stress, and other functions [73,88,91]. In the diabetes disease model, phytocomponents (ginsenoside Rg1, compound K, and a combination) and ginseng crude extract promote antidiabetic efficacies and alleviate associated side effects [92,93]. Moreover, in Type 2 diabetic patients, ginseng demonstrates potent antidiabetic efficacy, with no impact on prediabetes or healthy individuals [34]. Recently, Li and coworkers [94] have targeted the pyroptosis pathway in *P. ginseng* to improve efficacy in diabetes and related chronic complications, since the pathway (a form of programmed cell death) plays a critical role in the pathogenesis of diabetes. The article elucidated the role of the pyroptosis pathway and its mechanisms in diabetes and the need for targeting the pathway to establish the therapeutic efficacy of ginseng in clinical trials. These studies support further research on elucidating the molecular mechanisms of ginsenosides and screening new analogs with potent antidiabetic efficacies.

### 3.1. Antidiabetic Mechanisms of Ginsenosides and Clinical Efficacies

**Figure 2:**
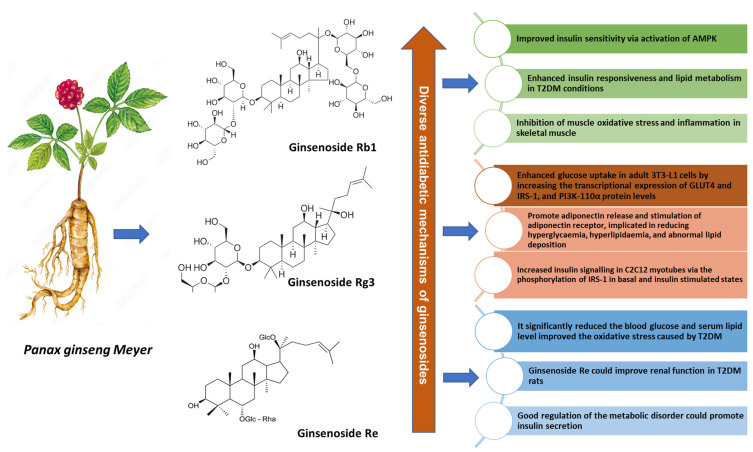
Representative examples of ginsenosides and their antidiabetic mechanisms in vivo. Ginsenoside Rb1, ginsenoside Rg3, and ginsenoside Re demonstrate potent antidiabetic efficacy in clinical trials, defining novel avenues in drug development.

Natural products are gaining increased momentum in the healthcare system due to their cost-effectiveness, remarkable efficacy, and minimal side effects. In animal models, ginseng demonstrates multiple anti-hyperglycemic mechanisms, comprising improved uptake of glucose (demonstrated by ginsenosides Rb2, Rg3, Re, Rc) [95,96,97], better insulin secretion or insulin sensitivity (demonstrated by ginsenosides Rb1, Rg3, Rg2) [98,99,100], liver glucose production (demonstrated by ginsenosides Rg5, Rg2) [101,102], restricted intestinal glucose absorption (demonstrated by ginsenoside Rg1) [103], and less accumulation of lipids (demonstrated by ginsenosides Rg1, Rg3) [104,105], (Figure 2).

Representative examples of ginsenosides and their antidiabetic mechanisms on animal models are discussed below:

#### 3.1.1. Ginsenoside Rg1 and Antidiabetic Mechanisms

Ginsenoside Rg1 is classified as a triterpenoid derivative of a tetracyclic monomer of the PPT class. Widely studied as a bioactive ginseng phytoconstituent, ginsenoside Rg1 demonstrates several pharmacological bioactivities and has few side effects [106]. Recent research discussed the potential of ginsenoside Rg1 to regulate glucose homeostasis and insulin sensitivity and alleviate hyperglycemia and associated complications [107]. Furthermore, ginsenoside Rg1 also significantly increased AMPK activity and increased glucose uptake via GLUT4 expression in differentiated C2C12 cells [97]. Subsequently, ginsenoside Rg1 induces the Akt pathway in the liver and primary hepatocytes in mice fed with an HFD, inhibiting hepatic gluconeogenesis and lowering fasting plasma glucose [108]. Studies have shown that ginsenoside Rg1 suppresses palmitate-induced insulin resistance in HepG2 cells, stimulates Akt, and prevents the production of ROS through the JNK pathway [109]. Moreover, ginsenoside Rg1 improved hepatic insulin resistance in mice (fed with a high-fat and high-sugar diet) by inhibiting inflammatory reactions and glucose production via activation of Akt signaling [110]. Through its antioxidant and anti-inflammatory effects, ginsenoside Rg1 exerts a beneficial effect on pancreatic cells, regulating blood glucose levels and inflammatory cytokines in hyperglycemic rodents [111]. Dong et al. [112] showed that ginsenoside Rg1 was injected into Type 2 diabetes mice (fed on HFD with streptozotocin), they showed significant improvement in cognitive dysfunction and neuronal injury, which indicates the promising therapeutic efficacies of ginsenoside Rg1 in Type 2 diabetes and in other diabetic complications. The ongoing studies on ginseng highlight the emerging clinical potential of ginsenoside Rg1 in disease management, particularly for Type 2 diabetes.

#### 3.1.2. Ginsenoside Rd and Mechanisms in Type 2 Diabetes

Ginsenoside Rd is primarily found in *P. ginseng*, and minor concentrations are present in *P. quinquefolius* and *P. notoginseng* [113] and are classified as the PPD-type saponins. Moreover, the different ginsenosides (including ginsenosides Rb1, Rb2, and Rc) can be further processed to ginsenoside Rd after absorption and metabolism in vivo [114]. In the present decade, pharmacological investigations on ginsenoside Rd have primarily focused on neuroprotective mechanisms, with recent findings suggesting that administration of ginsenoside Rd significantly alleviates hyperglycemia in streptozotocin diabetic rats (placed on a high-fat and high-sugar diet) [115]. The activation of the Akt signaling pathway by ginsenoside Rd in the liver promotes glycogen production and reduces hepatic glucose, ameliorating insulin resistance and obesity by stimulating thermogenesis and increasing glucose tolerance and insulin sensitivity in adipose tissue, skeletal muscle, and the liver [116]. Similarly, ginsenoside Rd has been demonstrated to reverse the effects of methylglyoxal on insulin desensitization and consequent apoptosis in primary astrocytes obtained from rats [117]. The anti-apoptotic mechanisms of ginsenoside Rd in Type 2 diabetes comprise hampered cell death and pro-apoptotic proteins in cultured human pancreatic islets [118]. He et al. [119] recently demonstrated that ginsenoside Rd reduces retinal endothelial injury at the high-glucose level, induces AMPK and Sirtuin 1 expression, and promotes their interaction. Taken together, these results highlight that the ginsenoside Rd demonstrates promising antidiabetic mechanisms and requires further investigation to assess its clinical prospects.

#### 3.1.3. Ginsenoside Rg3 and Antidiabetic Mechanisms

Another key example of a ginseng-specialized metabolite, ginsenoside Rg3, is present in a high concentration in KRG [120] and is further divided into two enantiomers, designated as 20(R) and 20(S), depending on the structure variation, based on the hydroxyl group at the C20 position, which explains its different pharmacological effects [120]. It has been widely researched for its antitumor, anti-inflammatory, antioxidant, anti-aging, and neuroprotective functions [121]. Recent findings have highlighted the therapeutic potential of ginsenoside Rg3 in multiple diseases associated with metabolic syndrome, like obesity and diabetes [122]. Interestingly, ginsenoside Rg3 remarkably enhanced glucose uptake in adult 3T3-L1 cells by increasing the transcriptional expression of GLUT4 and IRS-1 and PI3K-110α protein levels [123]. In addition, ginsenoside Rg3 directly interacts with PPARγ in adipocytes, promotes adiponectin release, and stimulates adiponectin receptors, implicated in reducing hyperglycemia, hyperlipidemia, abnormal lipid deposition, and dysfunction in adipose, liver, and heart tissues [124]. Furthermore, it increases insulin signaling in C2C12 myotubes via the phosphorylation of IRS-1 in basal and insulin-stimulated states [125].

Kim et al. [126] showed that ginsenoside Rg3 enhanced mitochondrial activity in C2C12 myotubes via upregulation of mitochondrial biogenesis genes, which might contribute to the improved insulin resistance of skeletal muscle. Similarly, ginsenoside Rg3 showed an anti-hyperglycemic effect on INS-1 cells (a rat insulinoma cell line) by triggering cell proliferation via stimulation of ERK and p38 MAPK signaling, which in turn reduced cell death under intermittent hyperglycemic conditions [126]. In line with this, ginsenoside Rg3 stimulated insulin release in hamster pancreatic HIT-T15b cells and streptozotocin-induced diabetic mice [127]. A recent study showed that ginsenoside Rg3 significantly promotes insulin secretion in pancreatic cells by enhancing GLP-1 secretion (e.g., NCI-H716 cells) in the Type 2 diabetic mouse model [99]. These studies highlight the positive antidiabetic mechanisms of ginsenoside Rg3 in addressing hyperglycemia, insulin resistance, and diabetes.

#### 3.1.4. Ginsenoside Rb1 and Mechanisms in Type 2 Diabetes

Ginsenoside Rb1 is classified as a PPD-type saponin and is mostly found in *P. ginseng*, *P. notoginseng*, and *P. quinquefolius* [128]. Ginsenoside Rb1 has low absorption in the intestine, but is subjected to intestinal microbial deglycosylation and is subsequently transformed into the secondary ginsenoside compound K through a stepwise hydrolysis reaction [128]. Studies have documented that ginsenoside Rb1 demonstrates multiple antidiabetic mechanisms in insulin resistance, hyperglycemia, and Type 2 diabetes. Ginsenoside Rb1 enhances the translocation of GLUT4 in 3T3-L1 and C2C12 cells by stimulating PI3K activity and IRS-1 and Akt phosphorylation [129]. Remarkably, the impacts of ginsenoside Rb1 on glucose uptake in the two cell lines appear to be partially mediated by the increase in the basal expression of adiponectin receptors (AdipoR1 and AdipoR2) and their signaling pathways [129]. Likewise, ginsenoside Rb1 positively affects insulin sensitivity via activation of AMPK [130], playing a partial role in restricted fat intake and inflammation in the liver [131]. Accordingly, administration of ginsenoside Rb1 to HFD-fed rats showed a significant decrease in body weight gain, body fat content, fasting glucose, and increased insulin sensitivity, as evidenced by the inhibition of gluconeogenesis in the liver and the increase in glucose uptake in skeletal muscle [131]. In obese (db/db) mice, ginsenoside Rb1 induced insulin sensitivity via decreased hepatic fat accumulation and adipocyte lipolysis [100]. Considering the integral association of insulin resistance and inflammation, treatment of mice with ginsenoside Rb1 significantly lowered inflammatory cytokines in the liver and adipose tissue and enhanced insulin responsiveness and lipid metabolism in obese and/or Type 2 diabetes conditions. Subsequently, ginsenoside Rb1 inhibited ER stress-induced inflammasome in adipose tissue, reduced the release of inflammatory cytokines, and alleviated insulin resistance [132]. Additionally, ginsenoside Rb1 inhibited muscle oxidative stress and inflammation in skeletal muscle in multiple Type 2 diabetes models [133,134]. Other mechanisms include ginsenoside Rb1 inhibition of pancreatic β-cell apoptosis (caused by high glucose) by hampering nitric oxide production and the expression of caspase-3 [135]. In general, ginsenoside Rb1 demonstrates favorable effects in the treatment of insulin resistance and Type 2 diabetes by increasing insulin sensitivity and overall metabolism, suggesting that it has clinical potential as an anti-hyperglycemic drug candidate [130]; however, further investigations are necessary to validate these findings.

### 3.2. Progress in the Evaluation of Ginsenoside-Based Antidiabetic Therapeutics in Clinical Trials

In the present decade, the pharmacological benefits of ginseng have been extensively investigated in preclinical and clinical trials to validate their therapeutic efficacy and prospects in diabetes management [136]. In addition to antidiabetic functions, ginseng demonstrates an anti-hyperlipidemic effect, suggested to be caused by the synergistic activities of the different phytoconstituents [137]. In a meta-analysis of 16 randomized controlled clinical trials, Shishtar et al. [138] demonstrated that ginseng had a significant lowering effect on fasting blood glucose compared to the control (−0.31 mmol/L [95% CI: −0.59 to −0.03], *p* = 0.03) but no effect on fasting plasma insulin and glycated hemoglobin [138]. A meta-analysis study (including eight trials) demonstrated ginseng supplementation showed significant differences on fasting glucose (−0.306 mmol/L [95% CI: −0.539 to −0.074], *p* = 0.01), postprandial insulin (−2.132 mmol/L [95% CI: −3.706 to −0.558], *p* = 0.008), and HOMA-IR (−2.132 mmol/L [95% CI: −3.706 to −0.558], *p* = 0.008) compared to the control group, while no significant difference in postprandial glucose and fasting insulin between the ginseng treatment group and the control group was observed. Since 2012, human clinical trials have investigated the antidiabetic efficacy of *P. ginseng*, *P. notoginseng*, and *P. quinquefolius*-based supplementations with variable outcomes in diabetic patients.

Park et al. [139] discussed that impaired fasting glucose participants were randomly given 960 mg/day hydrolyzed *P. quinquefolius* extract or placebo for eight weeks. Hydrolyzed ginseng extract including 7.54 mg/g ginsenoside Rg1, 6.30 mg/g compound K, 5.42 mg/g ginsenoside Rb1, 1.87 mg/g ginsenoside Re, 0.70 mg/g ginsenoside Rd, 0.36 mg/g ginsenoside Rb2, and 0.29 mg/g ginsenoside Rc, significantly lowered fasting plasma glucose (*p* = 0.017), and postprandial glucose levels (*p* = 0.01), but did not affect fasting plasma levels [139]. These findings provide key information that hydrolyzed ginseng extract (composed of different ginsenosides) reduces the intestinal absorption of glucose in the gut lumen and demonstrates antidiabetic efficacy, necessitating further research.

Lee et al. [140] showed that the ginseng berries comprise a different ginsenoside composition in a higher amount compared to ginseng roots. An in vivo study in ob/ob diabetic mice showed that administration of ginseng berry extract (150 mg/kg) significantly reduced fasting blood glucose levels from 150 mg/dL on day 5 to 129 mg/dL on day 12 [141]. Dey et al. [142] performed a comparative evaluation of the anti-hyperglycemic effects of ginseng root and berry extracts. Both the extract, ginseng root (150 mg/kg body weight), and ginseng berry (150 mg/kg body weight) significantly decreased fasting blood glucose to 143 +/− 9.3 mg/dL and 150 +/− 9.5 mg/dL on day 5 of administration. Moreover, while the ginseng berry extract (150 mg/kg body weight) considerably decreased body weight, the root extract (same concentration) did not affect the body weight [142]. The results showed a higher anti-hyperglycemic effect of ginseng berry extract, compared to ginseng root extract and ginseng-berry-mediated anti-obesity effects in ob/ob mice.

Choi et al. [143] performed a 12-week randomized, double-blind, placebo-controlled clinical trial on human participants (with fasting glucose levels between 100 and 140 mg/dL). The administration of ginseng berry extract decreased serum concentration of fasting blood glucose (by 3.7%) and postprandial glucose (at 60 min), assessed through an oral glucose tolerance test, demonstrating the potential to promote glucose metabolism. However, the anti-hyperglycemic effects of ginseng berry extract on lipid metabolism and blood glucose parameters were not found. Bang and coworkers [144] investigated KRG supplementation-mediated glucose regulation in human participants either recently diagnosed with Type 2 diabetes, IFG, or IGT conditions. A 12-week randomized, double-blinded, placebo-controlled or placebo trial was performed, and an oral glucose tolerance test was used to identify the glucose-related biomarkers (serum and whole blood levels of C-peptide, glucose, and insulin). A considerable decrease in serum levels of glucose and whole blood levels of glucose (at 30 min) was observed in the test group; however, no changes were seen in blood glucose-related indices (in the placebo group). In the test group, there was a significant decrease in C-peptide concentrations and serum insulin. The study showed that KRG administration (5 g/day) has a favorable effect on regulating whole blood glucose and serum levels in individuals with Type 2 diabetes, IFG, or IGT [144]. Cho et al. [145] evaluated the antidiabetic efficacy of KRG rootlets in a double-blinded, placebo-controlled, randomized trial. In the trial, 68 participants (BMI ≥ 23 kg/m^2^) received KRG or a placebo for a 12-week duration. The results showed that KRG did not improve insulin sensitivity in obese people who are non-diabetics.

In recent times, human trials based on ginseng-derived supplementation have garnered significant interest among the scientific community. Song and coworkers [146] performed a double-blind, randomized, and placebo-controlled trial including 1000 healthy individuals and evaluated the safety of KRG intake. The test group was administered KRG (2 g/day) for 24 weeks, and adverse drug reactions were reported at 4, 12, and 24 weeks. In the KRG and placebo groups, 192 and 211 individuals experienced adverse effects. While 59 and 57 KRG- and placebo-treated individuals showed adverse drug reactions, including diarrhea, dizziness, and headache, leading to discontinued administration in 13 participants in the KRG and placebo group. The study demonstrated the safety and tolerability of KRG extract with minor adverse effects on human participants [146]. In another study, AG and KGB were co-administered in human participants to evaluate the antidiabetic effect beyond conventional treatment methods [147]. In the randomized controlled trial, Type 2 diabetic patients (39 individuals) were registered in a hospital and kept on a constant diet, lifestyle, and medications. The ginseng-based supplementation (6 g of fiber from KRG and 3 g of AG) or wheat bran-based control was consumed by 30 participants for 12 weeks. After 12 weeks, a decrease in HbA1c levels and lipid levels was observed in the KGB-AG-treated group compared to the control, improving the effectiveness of the conventional medications [147]. Vuksan and coworkers [148] assessed the safety and efficacy of *P. quinquefolius* extract on cardiovascular risk factors and glycemic control in Type 2 diabetic individuals. The double-blind, crossover design study included 24 individuals who received *P. quinquefolius* extract (3 g/day) or placebo for 8 weeks, in continuation with the main treatment. The results showed that *P. quinquefolius* extract was effective in reducing fasting blood glucose (−0.71 mmol/L; *p* = 0.008) and HbA1c (−0.29%; *p* = 0.041) levels in the test group compared to the placebo. Moreover, the plant extract decreased systolic blood pressure and increased serum nitrates/nitrites but did not alter safety profiles [148]. In a clinical trial on *P. ginseng*, 36 diabetic patients received 1.5 g/day of ginsam (high concentration of ginsenoside Rg3) via daily supplementation for 8 weeks. Ginseng administration in individuals showed a significant decrease in HbA1c levels (0.56%) and fasting blood glucose (21.40 mg/dL) compared to the placebo group [35] and reduced glucose absorption in the intestine by hydrolyzed ginseng extract. The growing evidence on ginseng-based human trials validates the antidiabetic potential of different plant species in the genus *Panax*; however, further research is crucial to consolidate the experimental findings and bridge the knowledge gaps.

## 4. Discussion

**Nano-Ginseng and Breakthroughs in Drug Development: Addressing Therapeutic Bottlenecks via Nanobiotechnology**

Nanotechnology-driven innovations have made a significant contribution to natural product-mediated drug development. In the treatment of diabetes, patient compliance is a key consideration, considerably improved by nanoformulation approaches through multiple routes of drug administration, controlled and targeted release, improved stability, and the reduced toxicity of natural products [50,53,149]. Nature-derived medicines comprise a principal share in the drug market, with plant-based medicines showing promise and potential efficacy in diabetes and associated complications [150,151,152]. A paradigm shift in the development of nano-based antidiabetic formulations is attracting considerable interest from the global scientific community. Phyto-nanomedicines have been instrumental in improving the biopharmaceutical attributes of herbal drugs, which are clinically comparable to those of commercial antidiabetic medicines [153]. In addition, improved therapeutic properties, including stability, pharmacokinetics, and better efficacy of green-synthesized NPs (silver, gold, and zinc oxide nanoformulations) have been pivotal in diabetes management [153].

**Figure 3:**
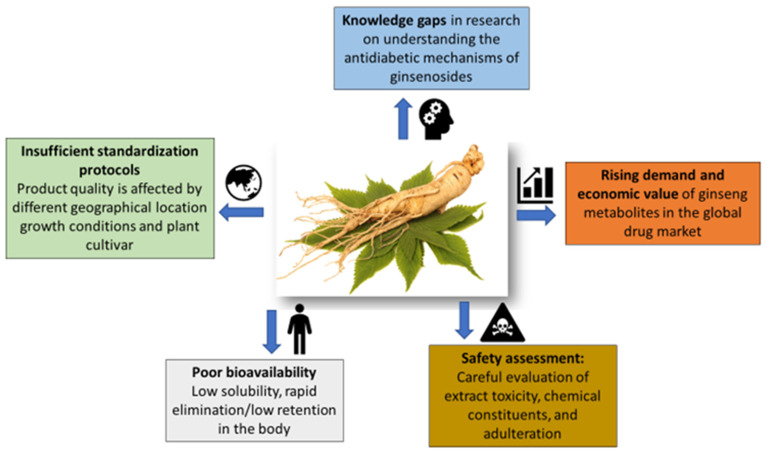
Major bottlenecks in the development of ginseng-derived antidiabetic therapeutics.

Among medicinal plants, the *Panax* genus (family *Araliaceae*) has a rich and traditional pharmacological history of treating multiple human ailments. In diabetic disease models, ginsenoside Rg1, compound K, and their combined form, as well as crude plant extract, have demonstrated synergistic effects, improved antidiabetic efficacy, and have few side effects [93,154], showing promising outcomes. Despite potent bioactivities, the poor pharmacokinetics of ginsenosides hinder further evaluation in clinical trials [155,156], possibly due to their interaction with cell membranes, ion channels, and receptors caused by transcriptional changes, cytotoxicity, and poor solubility [157,158,159]. Studies have further documented the elimination of ginsenosides by efflux transporters, causing poor absorption and hampering further research [160,161] (Figure 3).

Of the 17 *Panax* species reported globally, *P. ginseng*, *P. notoginseng*, and *P. quinquefolius* are widely recognized for their therapeutic significance and are used in the synthesis of diverse nanoparticles and their applications. Progress in ginseng nanobiotechnology has been instrumental in enhancing ginsenoside properties and addressing the poor bioavailability of phytoconstituents through nano-encapsulation or nano-sizing [92]. Nano-sizing of phytoconstituents facilitates BBB permeability, regulates insulin production, and enhances brain glucose supply [162]. The potential of nano ginseng in diabetes offers innovative solutions, including the use of ginseng saponins (derived from the fruit) for proliposome synthesis and sodium deoxycholate, which demonstrate improved delivery and highlight promising therapeutic applications [92]. In addition, innovative drug-delivery approaches, including micellar formulations, polymer–drug conjugates, and nanoparticle encapsulations, are being developed for ginseng metabolites [163]. These advanced nano-innovations are designed to address the existing challenges of solubility, stability, circulation time, and the targeted and controlled release of ginsenosides. The unmatched versatility of polymeric carriers facilitates optimization of molecular weight and chemical composition and allows adjustment of functional groups to directly control drug loading capacity, release kinetics, and targeting [164]. Most polymers used in drug delivery (e.g., PLGA, chitosan, PEG) are biocompatible and biodegradable and may be regulated in medicinal applications. In addition, biological barriers can be countered by these polymeric systems via targeted ligand attachment, stimuli responsiveness (response to changes in pH, temperature, or redox conditions), biomimicking through cell membrane coatings [165], and scalable production. While nanocarrier-mediated delivery of the polyherbal formulation clearly indicates its efficacy in decreasing blood sugar levels, investigations of medicinal plants and phytoconstituents are still required to evaluate their individual potential for Type 2 diabetes. While nanocarriers may be engineered for reaching targeted cells or tissues, the encapsulated drugs are delivered through nanocarrier degradation; however, the stability and degradation of nanocarriers in biological systems require further investigation [166]. Furthermore, metal oxide or inorganic metal nanocarriers have several benefits in therapy and imaging; however, challenges with slow degradation, toxicity, and rapid elimination hamper further medical applications [167].

**Figure 4:**
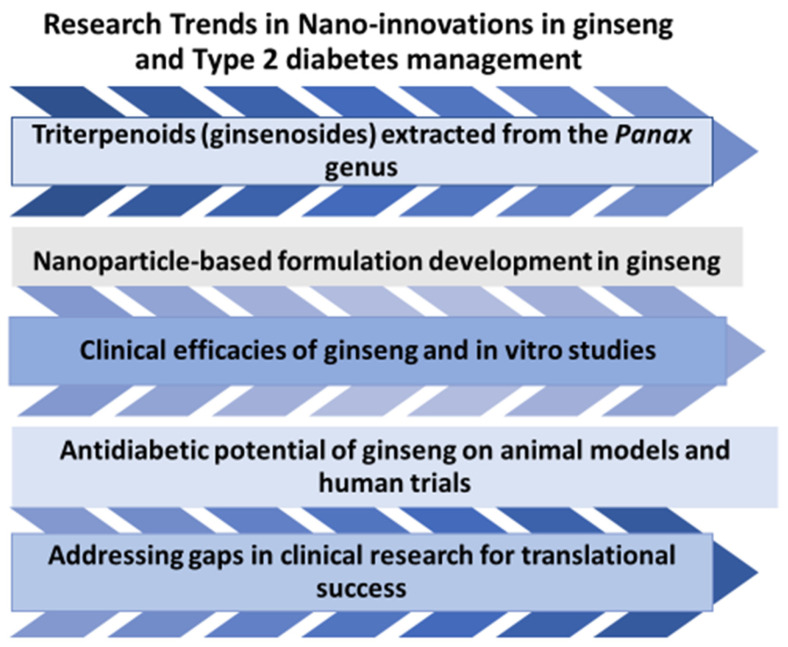
Research trends in the development of nano-ginseng for Type 2 diabetes. The schematic overview shows nanotechnological interventions in ginseng to improve pharmacokinetics and facilitate drug development.

In a key study, targeted and precise activity of ginseng phytomolecules was observed via nanocarrier delivery systems in multiple animal models [168]. Patil et al. [169] developed a polyherbal formulation composed of *Momordica charantia*, *P. ginseng*, and *Cinnamomum verum* by entrapping the bioactive constituents into nanocarriers (liposomes, polymeric NPs, and solid NPs). The nanocarrier-based polyherbal formulations remarkably improved the sustained release and bioavailability of the bioactive constituents. Furthermore, the polyherbal composition substantially reduced blood glucose levels in diabetic rats, demonstrating prominent glucose-lowering effects [169]. Ongoing investigations of ginseng provide strong evidence that the phytoconstituent ginsenosides Rg1, Rd, Rg3, and Rb1 demonstrate potent efficacy in alleviating symptoms of Type 2 diabetes and its associated complications. Research on ginseng-based nano-innovations for Type 2 diabetes is still in its early stages, and there is a lack of clinical studies on the development of ginseng nanoformulations. Most of the current research evidence is supported by in vitro studies; further studies are necessary to validate these findings and bridge the existing knowledge gaps (Figure 4).

### Considerations of Safety, Toxicity, and Regulatory Issues of Nanomaterials in Healthcare Applications

While the application of nanotechnology has potential benefits and is fast gaining recognition, key concerns with biocompatibility (safety), environmental impact, and current regulatory policies are crucial and require attention. When a foreign particle, such as a nanomaterial, is administered in the human body, a reduced particle size (nanosizing) shows novel attributes; however, it can also cause unconventional toxicity. In addition, improper exposure to nanomaterials can cause medical conditions and prolonged damage to human tissues [170]. Therefore, it is essential to assess the human health risk via the life cycle assessment of the product and its environmental impact, including its potential negative impact on human health [171]. Moreover, the assessment of nanomaterials for environmental and ecological safety necessitates the development of new approaches. Globally, current regulatory policies are actively involved in defining the regulation and standardization of nano-based products [172]. Presently, China, Europe, and the United States have launched initiatives for the evaluation and regulation of nanomaterials. The Environmental Protection Agency (EPA) of the United States successfully implemented guidelines for environmental and health safety research on nanomaterials, particularly on their transformation, distribution in the environment, and toxicological characteristics. Similarly, the Scientific Committee of the European Commission issued guidelines on biosafety and environmental assessments of synthetic nanomaterials. The regulatory concerns about nanomaterial use focus on factors such as size, distribution, surface modification, whether they are natural or synthetic, durability, and other properties [172]. Subsequently, the European Parliament’s REACH framework defines a framework for the safety assessment of nanomaterials and an evaluation of their hazards. Risk assessment of nanomaterials, aligned with regulatory guidelines, would improve risk management approaches and facilitate a more careful application of nanotechnology in human health.

## 5. Conclusions

The projected global disease burden substantially impacts the world’s economies, with Type 2 diabetes and its associated complications predicted to have a high prevalence. Recent biotechnological progress in therapeutic development has witnessed remarkable breakthroughs, including natural-product-based combination/repurposing therapies, nanotechnological advances, and precision medicine [15,173]. While these demonstrate their own benefits and limitations, novel medications with specificity, improved drug delivery mechanisms, and better safety profiles are urgently required in drug development. In this direction, the development of nano-based herbal medicines, including herbal nanoformulations and their targeted and precise delivery via nano-delivery systems, has been remarkably successful in addressing the bottlenecks associated with herbal antidiabetic drugs [36,37].

Some success stories in nanotechnology-based drug development for diabetes include Zhang et al. [174], who discussed the development of biotin-based liposome modification as an effective strategy for oral insulin delivery and improved insulin uptake via the intestinal epithelium. The nanoliposomes demonstrate higher tolerance to enzymatic degradation; therefore, they have a longer retention time in the gastric tract. Sheng and coworkers [175] linked insulin with peptide-protamine, encapsulated in PLGA NPs with an N-trimethyl chitosan chloride coating. The nanoformulation demonstrated improved bioavailability and an instant hypoglycemic effect (for longer durations) when administered in diabetic rats [175]. Furthermore, SLNs (as nano-based drug delivery systems) showed potential to improve intestinal absorption and prevent enzymatic degradation of hypoglycemic agents [176]. In preclinical models, remarkable improvement in therapeutic efficacy and pharmacokinetics was achieved by metformin-loaded alginate nanocapsules, repaglinide-loaded polymeric systems, and glipizide sustained-release NPs. These nano-interventions highlighted improved oral bioavailability, better glycemic regulation, and a low dose requirement for diabetes [177,178]. Rao et al. [179] emphasized emerging trends in the use of ML tools for designing novel nanotheranostics, saving time and result analysis, and offering key opportunities in ML-based nanotheranostics for disease management. Moreover, ML-based nanotheranostics projects clinical benefits to the patients via advanced ML models and reliable datasets, expanding therapeutic horizons. In this field, plant-based nanotechnological advances are pivotal and focus on improving the antidiabetic efficacy and pharmacokinetics of phytoconstituents through the development of plant-based nanoformulations.

In recent times, the *Panax* genus has gained significant recognition, attributed to the promising pharmacological properties of the plant species. In the global market, ginseng has an economic value of USD 2.1 billion [180] and is sold as ginseng powders, teas, capsules, and plant roots. With a rapidly expanding market, ginseng-derived products and formulations comprise a significant economic share; however, careful consideration and regulatory guidelines are necessary to assess and prevent increasing adulteration, adversely impacting the economic value of the marketed products. The increased recognition of ginsenosides in Type 2 diabetes has been pivotal and defines novel avenues in drug development. Several in vivo studies have clinically demonstrated the anti-hyperglycemic effects of ginsenosides Rb2, Rg3, Re, Rc, and Rg5 via mechanisms that include improved insulin secretion or insulin sensitivity, enhanced glucose uptake, reduced intestinal glucose absorption, decreased liver glucose production, and reduced lipid accumulation. As is evident from clinical trials, ginsenosides Rb1 and Rg1 demonstrate potent antidiabetic mechanisms in Type 2 diabetic patients. However, discrepancies between preclinical and clinical trials are observed, which may be due to a small participant group, the complex chemical structure of ginsenoside causing poor bioavailability, or inaccurate study designs, requiring further investigation [92]. To maintain the therapeutic activity of specialized metabolites (as evident in preclinical studies), novel approaches should focus on the modulation of drug dosage forms, gut microbiota-driven biotransformation, and the solubility of phytoconstituents [136].

Nano-innovations in ginseng have the potential to address current therapeutic challenges and improve systemic circulation, reduce toxicity, and improve bioavailability, facilitating natural-product-mediated drug development. In addition, co-administration of two or more ginseng compounds with conventional therapeutics for improved efficacy or drug repurposing is another promising strategy to improve antidiabetic efficacy and disease management. Although the prospects and future possibilities are interesting, there is currently a lack of clinical studies on ginseng-based nanoformulations for Type 2 diabetes, with key information obtained from animal models. To validate these findings, well-designed human clinical trials are essential to evaluate the antidiabetic potential of ginseng and/or ginsenosides. While nano-based interventions of ginseng for diabetes management are in the initial stage and starting to gain recognition, extensive research is necessary to facilitate bench-to-clinical translation: criteria concerning the quality and safety of herbal drugs, safety assessments, bioactivity investigations, analytical methods employed for product characterization, and preclinical validations are essential. Other considerations of upscaling and production require regulatory compliance and a robust manufacturing process to achieve the desired translational success.

## Figures and Tables

**Table 1:** Nanobiotechnology-driven innovations in diabetes and associated complications.

Smart Nanotechnologies	Innovative Approaches in Diabetes Healthcare	Mechanisms in Type 2 Diabetes and Associated Complications	Translational Success/Bottlenecks	References
Diabetes diagnostics and treatment (theranostics)				
Nanosensor application for diagnosing diabetes and management	Periplasmic ligand-binding proteins as glucose nanosensors	The developed glucose nanosensors were more robust, used new reporters, and were responsive within the physiological range of blood glucose levels	The developed glucose nanosensors were more robust and efficient for measuring glucose levels	[48]
	For subcutaneous delivery of glucose, HMSNs with dual-responsive copolymer coatings were developed	The glucose-mediated MN device showed glucose adjustment and drug release	A rapid drug release at high blood sugar levels, while drug release was delayed at the normoglycemic level, showing a controlled drug release in diabetes management	[49]
	CeO_2_ NPs for gestational diabetes treatment	Nanoceria (60 mg/kg) prevented mitochondrial toxicity and damage	Prevents mitochondrial damage due to gestational diabetes	[50,51]
	The resveratrol–zinc oxide complex is encapsulated for gestational diabetes	Chitosan-encapsulated nano resveratrol minimized the side effects in resveratrol delivery and increased bioavailability	The levels of endoplasmic reticulum stress (GRP78, p-IRE1α, p-eIF2α, and p-PERK) and inflammation factors (IL-6 and MCP-1) were decreased, and blood glucose levels were substantially reduced	[52]
	RME loaded on polyacrylic gold nanoparticles	In diabetic mother rats, liver tissues showed remarkable improvement. Au-PAA-NPs extract regulates serum glucose levels	In the new method, AuNPs were effectively used for controlled and precise drug delivery	[53]
Nano drug-delivery systems for diabetic wound healing				
Nanocarrier-based solutions for wound healing in diabetes	AuNPs-mediated delivery of siRNA (PHD-2 downregulation)	PHD-2 silencing, pro-angiogenic pathways upregulation	Improved diabetic wound healing with siRNA therapy	[54]
	Nanofibers composed of *Malva* *sylvestris*-neomycin sulfate	*M. sylvestris*-loaded nanofibers showed better antibacterial activity, potent efficacy, and reduction in acute and chronic inflammation	Improved wound healing through the use of plant-based nanofibers	[55]
	Nano-in-micro hydrogels (microbeads), CCMBs containing Cur-R, were developed	Cur-R-CCMBs showed good antibacterial activity against MDR wound pathogens, higher anti-inflammatory and antioxidant activity	Nano-in-micro hydrogels improve the efficacy of hydrophobic antimicrobials against resistant pathogens associated with wound infections	[56]
	HA-PEI nanoparticle-based delivery of siRNA-29a gene	Production of angiogenesis factors (α-SMA and CD31) and pro-inflammatory factor inhibition	In diabetic wound treatment, hyaluronic acid-based hydrogel with miRNA-laden nanoparticles improves anti-inflammatory response and angiogenesis	[57]
	Nanoformulation-mediated delivery of CCN1	Reduce inflammation, promote CCN1 expression	The topical application of CCN1-NP promoted wound healing in both in vitro and in vivo conditions	[58]
	Chitosan hydrogels were used for L-glutamic acid delivery	Promote macrophage activity and vascularization	Fast healing of diabetic wounds through improved angiogenesis and deposition of collagen	[59]
Nanotechnology in oral administration of insulin				
Nano-innovations in oral insulin formulations	Polysaccharide-mediated insulin delivery	Good biocompatibility and protein affinity	Safe and efficient insulin delivery	[60]
	Nanogels composed of concanavalin A crosslinked to glucomannan	Regulation of blood glucose levels	Effective and controlled drug release protects drugs from enzymatic degradation	[61,62]
	A polyelectrolyte coating of W/O/W nano-lotion containing Insulin was developed	Improves oral relative bioavailability and changes drug release characteristics	Prolonged release of oral insulin dosage forms	[63]
	Polymer nanospheres/capsules (chitosan, alginic acid, hyaluronic acid)	Enhanced oral insulin delivery	Considerable reduction in blood glucose levels	[64]
	Liposome-mediated oral insulin delivery (L-arginine, sodium alginate)	Protects against enzyme degradation, cell-specific targeting, and improves the drug solubility	Precise and controlled insulin release, improved oral bioavailability	[65]
Nano-innovations in Gene Therapy for Type 2 diabetes				
Gene therapy progress	Delivery of glucagon receptor siRNA using lipid NP technology	Decrease in blood glucose levels, plasma glucagon increases	Glucose homeostasis improves in mouse models of diabetes	[66]
	Lecithin-based nano-liposomal carrier to target diabetes associated genes	DPP-4 gene expression blocked, normalized blood glucose levels, and reduced kidney and liver damage	The therapeutic Cas9-RNP-based nano-liposomal carrier system benefits genome editing therapies	[67,68]
	siRNAs encapsulated within glucan microspheres	Silences genes in inflammatory phagocytic cells	Modifies lipid synthesis in hepatic cells in patients with metabolic syndrome	[69]
	Plasmid DNA (encoding IL-4 and IL-10), loaded in poly[gamma- (4-aminobutyl)-L-glycolic acid] NPs	Suppression of insulitis, immune regulation by delivery of a therapeutic gene	Prevention of diabetes	[70]
Plant-based nanoformulations for Type 2 diabetes treatment				
Plant-based nanoformulations	Lycopene-loaded Niosomes	A significant reduction in blood glucose level, a reduction in total cholesterol levels	Increased hypoglycaemic activity, antihyperlipidemic activity	[71]
	Polymeric nanoformulation of GL and TQ	Significant improvements in body weight and lipid profile, reduction in HbA1c and blood glucose levels	Increased antidiabetic activity, low cytotoxic effects	[72]
	Baicalin-loaded NLCs	Sustained and controlled release of Baicalin from NLCs	Higher antidiabetic activity of the nanoformulation	[73]
	Myricitrin-based SLNs	Myricitrin SLNs showed antioxidant, antidiabetic and anti-apoptotic effects	Improved hyperglycemia complications and diabetes	[74]
Nanosensors and glucose monitoring in diabetes				
Wearable and implantable nanosensors	Wearable glucose sensors	Detection, measurement, and monitoring of biofluid biomarkers	Glucose monitoring and regulation in body fluids for managing diabetes	[75]
	Glucose oxidase enzyme-based biosensors	Withstands a wide range of pH and temperature, selective for the glucose target	Glucose management, affordable and convenient to use	[76]
	Skin-worn glucose biosensors	Non-invasive glucose monitoring	Improved glycemic control	[77]
	Implantable nanosensors (The Eversense CGM System)	An implantable needle-style sensor, transmitter, and display monitor	Long-term glucose monitoring	[78]

## Data Availability

No new data were created or analyzed in this study. Data sharing is not applicable to this article.

## Abbreviations

DM	Diabetes mellitus
WHO	World Health Organization
GLP	glucagon-like peptide-1
IGT	impaired glucose tolerance
KRG	Korean red ginseng
NHDs	nanotized herbal drugs
GLP-1 RA	glucagon-like peptide-1 receptor agonist
SGLT2	sodium-glucose cotransporter 2
ROS	reactive oxygen species
PTP1B	protein tyrosine phosphatase 1B
PPARγ	peroxisome proliferator-activated receptor γ
TC	total cholesterol
VLDL	very-low-density lipoprotein
HDL	high-density lipoprotein
LDL	low-density lipoprotein
ML	machine learning
NPs	nanoparticles
NLCs	nanostructured lipid carriers
HMSNs	hollow mesoporous silica nanoparticles
CeO_2_	cerium oxide
CS-ZnO-RS	chitosan-zinc oxide-resveratrol
GRP78	glucose-regulated protein 78
p-IRE1α	phospho-inositol-requiring enzyme 1 alpha
p-eIF2α	phospho eukaryotic initiation factor-2α
p-PERK	phospho protein kinase R (PKR)-like endoplasmic reticulum kinase
IL-6	interleukin 6
MCP-1	monocyte chemoattractant protein-1
RME	*Ramulus mori* extract
AuNPs	gold nanoparticles
siRNA	small interfering RNA
PHD-2	prolyl-hydroxylase domain 2
CCMBs	chitosan–carrageenan microbeads
α-SMA	α-smooth muscle actin
curcumin-loaded rhamnosomes	platelet endothelial cell adhesion molecule 1
CD31	Cur-R
CCN1	cellular communication network factor 1
DPP-4	dipeptidyl peptidase-4
Cas9-RNP	CRISPR-associated protein 9-ribonucleic protein
DNA	deoxyribonucleic acid
GL	glycyrrhizin
TQ	thymoquinone
SLNs	solid lipid nanoparticles
GOx	glucose oxidase
PLGA	poly(lactic-co-glycolic) acid
HbA1c	hemoglobin A1c
HPLC	high-performance liquid chromatography
MS	mass spectrometry
USD	US dollar
PRISMA	preferred reporting items for systematic reviews and meta-analyses
PPT	protopanaxatriol
PPD	protopanaxadiol
AMPK	AMP-activated protein kinase
GLUT4	glucose transporter type 4
C2C12	mouse myoblast cell line
ERK	hepatocellular carcinoma extracellular signal-regulated kinase
p38 MAPK	p38 mitogen-activated protein kinase
HepG2	hepatocellular carcinoma cell line
AKT1	serine-threonine protein kinase
JNK	c-Jun N-terminal kinase
IRS-1	insulin receptor substrate 1
PI3K	phosphatidylinositol 3-kinase
HFD	high-fat diet
HOMA-IR	homeostasis model assessment of insulin resistance
IFG	impaired fasting glucose
BMI	body mass index
AG	American ginseng
KGB	konjac-glucomannan-based fiber blend
BBB	blood–brain barrier
PEG	polyethylene glycol
